# Stimulated Cooling of a Frenkel Exciton-Polariton Bose–Einstein Condensate at Room Temperature

**DOI:** 10.1021/acsphotonics.5c03069

**Published:** 2026-04-22

**Authors:** Thomas M. Khazanov, Cora A. Noble, Andrew J. Musser

**Affiliations:** Department of Chemistry and Chemical Biology, 5922 Cornell University, Ithaca, New York 14853, United States

**Keywords:** exciton-polariton, polariton Bose−Einstein condensation, thermalization, lasing, stimulated cooling

## Abstract

Exciton-polaritons have been widely studied for their ability to form a lasing state at sufficiently high excitation density. These states are typically described in terms of Bose–Einstein condensation, but in driven-dissipative systems, they achieve thermal equilibrium only in exceptional cases. Explicit consideration of the nonequilibrium nature of polariton condensates suggests that under incoherent pumping, they can achieve thermalization below the bath temperature. We provide experimental support for this effect in a simple Fabry–Pérot architecture exploiting the unique physical characteristics of organic semiconductors. We find that the effective temperature and chemical potential of the polariton gas can be tuned by pumping rate, enabling significantly sub-bath cooling of the polariton population. This effect occurs spontaneously, with no need for external potentials, and arises from the rapid thermalization and confined Frenkel excitons of the organic active layer. Furthermore, we show that exciton leakage through the cavity arising from the large-bandwidth emission of conventional organic semiconductors provides a unique probe of the underlying exciton photophysics, such as the efficiency of bosonic stimulation, inaccessible to semiconductors with narrow emission line widths.

## Graphical abstract

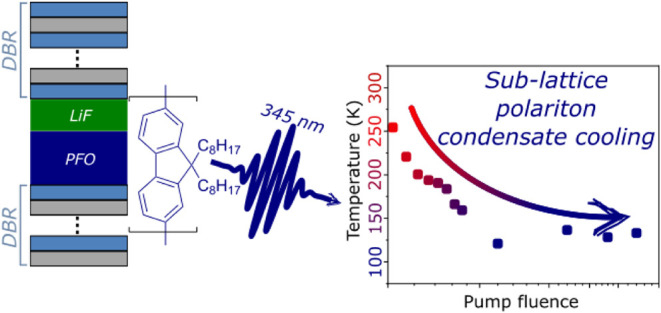

## Introduction

Exciton-polaritons are bosonic quasiparticles, resulting from the strong coupling of excitons to the electromagnetic field. As bosons, exciton-polaritons—henceforth, polaritons—are expected to undergo Bose–Einstein condensation at a critical temperature or density.^1^ Though the concept of polariton has been known in the literature since at least as early as the 1950s,^2–4^ polariton Bose–Einstein condensates (BECs) were only unambiguously observed in 2006.^5^ First reported in a Fabry–Pérot cavity containing CdTe/CdMgTe quantum wells at cryogenic temperatures, polariton BECs have since been observed in a variety of systems at temperatures as high as hundreds of kelvin, employing excitonic materials such as perovskites,^6,7^ transition metal dichalcogenide monolayers,^8,9^ semiconductor nanocrystals,^10^ molecular materials,^11–13^ semiconducting polymers,^14^ dyes in solution,^15^ and even fluorescent proteins.^16–19^ To date, a number of interesting developments have been reported, such as polariton transistors and logic gates,^20–22^ Hamiltonian simulators,^23–25^ observation of vorticity^26–30^ and superfluidity,^31–33^ and large optical nonlinearities even at the single photon limit.^34^

These effects have been typically described in the context of BEC physics, though there has been debate about whether this is an appropriate description, particularly in the case of organic materials.^1,17,35^ Typically, BECs have been considered at thermodynamic equilibrium at ultracold temperatures, such as in the case of a gas of ^87^Rb atoms.^36^ As open dissipative systems, semiconductor microcavity polaritons thus necessarily exist far from thermodynamic equilibrium.^37^ The polariton population must be maintained by external pumping, the removal of which results in rapid dissipation of the BEC.^19,38^ Because of this, terms such as polariton condensation, polariton lasing, BEC, and nonequilibrium BEC have all been used to describe the same set of observed phenomena in polaritonic systems (typically, some combination of nonlinear increase in emission intensity, nonlinear decrease in line width, emission blueshift, and increased spatiotemporal coherence), raising the question of what is and what is not, in fact, a BEC. This is further complicated by the fact that many of these same effects also occur during photon lasing, resulting in some ambiguity as to which systems genuinely exhibit condensation.^39,40^

Recently, Shishkov et al. reported an analytical solution for the density matrix of a nonequilibrium polariton BEC based on a Lindbladian approach applicable when polariton thermalization is fast and polariton interactions are small.^37^ From the density matrix, the polariton population can be obtained as a function of two key parameters: (1) the ratio of the incoherent pumping rate of the exciton reservoir to its dissipation rate κ/γ, and (2) the frequency ratio ℏ­(ω_*j*_ – ω_0_)/*k*_B_ *T* for each state, where ℏ­(ω_*j*_ – ω_0_) is the energy of state *j* relative to the lowest energy polariton state, *k*_B_ is the Boltzmann constant, and *T* is the temperature. The calculated polariton distributions were fit to an equilibrium Bose–Einstein distribution of the form

1 $$\overset{®}{n_{j}\;}=A{\left(e^{\left(\hbar \left(\omega_{j}-\omega_{0}\right)-\mu \right)/k_{B}T}-1\right)}^{-1}$$

where $\overset{®}{n_{j}\;}$ is the average particle number of the *j* ^th^ state, μ is the chemical potential, and *A* is a constant. From this, the population as a function of the temperature and chemical potential as described by eq 1 of the polariton gas can be approximated. Above the threshold density, μ approaches zero as expected (Figure S1).^37^ Interestingly, above the threshold, the polariton temperature is expected to collapse well below the bath temperature, resulting in stimulated cooling of the polariton gas (Figure S1). This is in contrast to BECs at thermodynamic equilibrium and is a result of the nonequilibrium nature of polariton BECs. Furthermore, this stimulated cooling behavior is observed in the absence of a trap or other external potential—distinguishing it from evaporative cooling—and represents a potential advantage of organic materials where an efficient cooling mechanism is required.

Herein, we aim to explore this theory experimentally. We have chosen the semiconducting polymer polyfluorene (PFO) as the active material since condensation has already been well characterized in PFO.^14^ Additionally, organic materials have been reported to exhibit fast polariton thermalization rates, as well as negligible polariton–polariton interactions due to the small Bohr radius of Frenkel excitons, thus satisfying the conditions laid out by Shishkov et al.^37,41–44^ Furthermore, the large exciton binding energy of Frenkel excitons allows for the observation of condensation, even at room temperature. We report the stimulated cooling of the polariton gas above the condensation threshold, as observed experimentally, and discuss its implications for polariton condensation.

## Experimental Methods

The PFO used in this work was purchased from Ossila. It was dissolved in toluene at approximately 10 mg/mL and stirred at 70 °C for an hour. To obtain amorphous films, the PFO solution was spin-coated onto quartz substrates while still hot. DBRs were fabricated by radiofrequency magnetron sputtering of alternating layers consisting of 50 nm of TiO_2_ and 72 nm of SiO_2_. The final cavity structure consisted of a bottom DBR of 9.5 pairs deposited on quartz, the PFO active layer, a thermally evaporated LiF layer, and a top DBR of 7.5 pairs.

Photoluminescence spectroscopy measurements were performed with a Light Conversion Pharos 10W femtosecond pulsed laser (1030 nm, 8 kHz, 180 fs) as the excitation source and measured with a Princeton Instruments Spectrapro HRS300 spectrometer (150 g/mm) and a PI-MAX4 ICCD camera. During alignment, the sample position was determined by optimizing the observed emission to correct for chromatic aberration of the objective. Excitation fluences were estimated by sending an excitation beam of the same wavelength as the sample emission to the sample stage, where a beam profiler was placed. The distance was adjusted until the beam was measured at its focal point, at which point a beam of the desired excitation energy was sent to the profiler, and the beam diameter was measured. Average power was measured by using a power meter. The fluence was then calculated using the measured beam diameter and power, accounting for transmission through the top DBR of the cavity at 10° to normal incidence. Data were processed and analyzed by using MATLAB and Origin.

## Results and Discussion

Fabry–Pérot cavities containing a neat film of PFO between two distributed Bragg reflectors (DBRs) were fabricated as described in the experimental methods. The absorption spectrum of PFO exhibits an inhomogeneously broadened excitonic feature at 3.25 eV, consistent with the glassy amorphous form of the polymer. This is in contrast to the emission spectrum, which exhibits a partially resolved Franck–Condon progression of vibronic replicas at ca. 2.90 and 2.75 eV. This is typical of semiconducting polymers, as polymer excited states generally tend to be more ordered and planar relative to the ground state, and thus exhibit more structured emission spectra.^45^ The DBR stopband (Figure 1a) was designed to maximize overlap with the absorption and emission spectra while maintaining high transmission at higher energies to allow excitation of the microcavity through the top DBR. The full cavity structure consisted of a 9.5-pair DBR deposited on a quartz substrate, followed by a spin-coated active layer of PFO, a thermally evaporated layer of LiF, and finally a 7.5-pair DBR as described in Figure 1b. The LiF acts as a spacer layer as well as a protecting layer for the PFO during sputtering of the top DBR. Based on transfer matrix modeling^46^ (TMM) of the corresponding cavity photon mode (Figure S2), we estimate a theoretical upper-limit Q-factor of the cavity to be approximately 2500. While the experimentally achieved Q-factor is likely to be lower than this value, our observation of hallmarks of polariton BEC (see below) indicates that the Q-factor should be moderately high.

**Figure 1:**
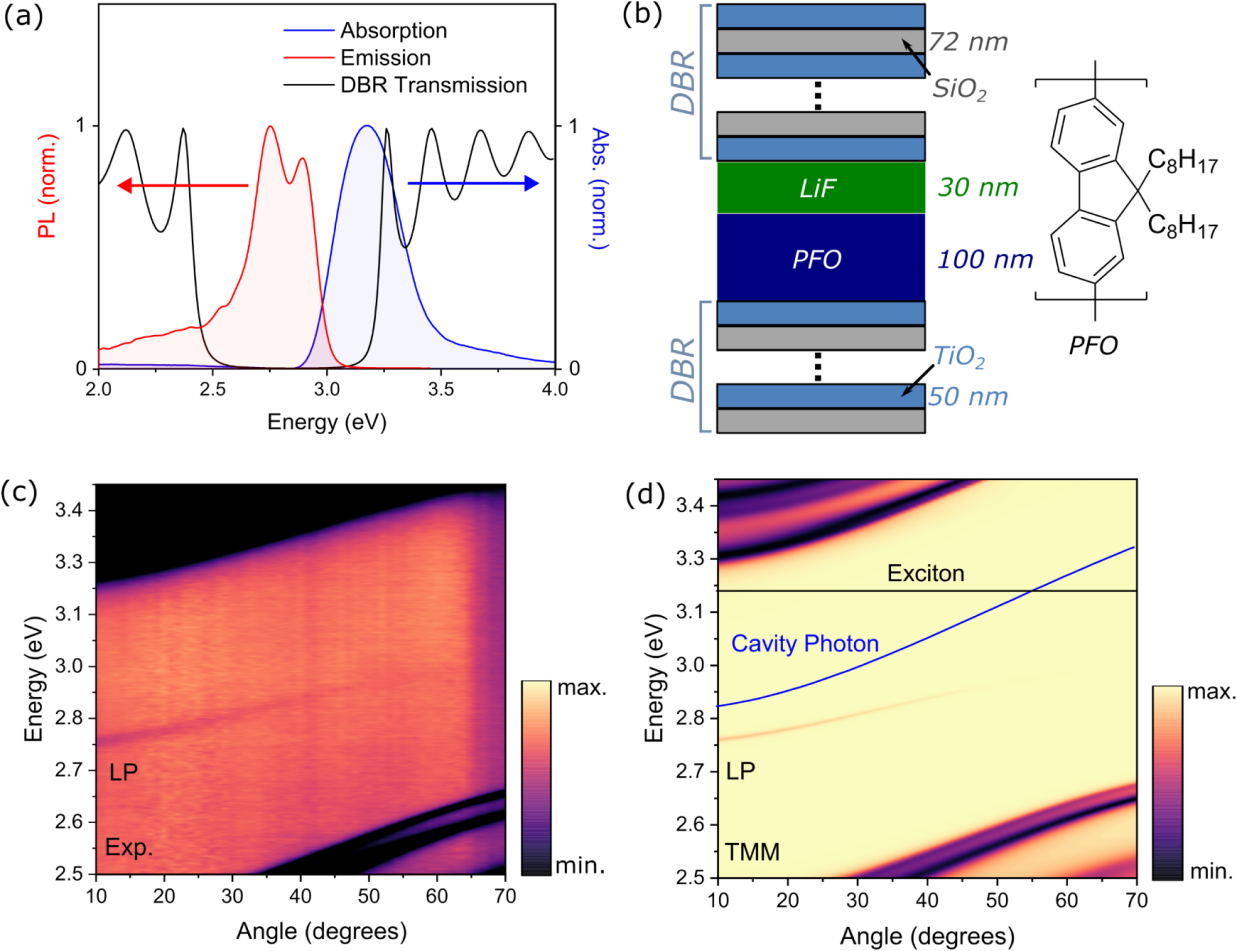
(a) Normalized absorption (blue) and emission (red) spectra of a thin film of PFO, and normalized DBR transmission spectrum at normal incidence (black). (b) Left: Fabry–Pérot cavity schematic; right: structure of PFO. Bottom DBR: 9.5 pairs; top DBR: 7.5 pairs. (c) Experimental *s*-polarized angle-resolved reflectivity spectra of the cavity in (b). (d) Transfer matrix model of the experimental *s*-polarized reflectivity map in (c). The cavity photon mode from the transfer matrix model and the PFO exciton energy are superimposed in blue and black solid lines, respectively. The transfer matrix model was parametrized by strong-coupled metallic cavities exhibiting both LP and UP modes, and PFO-free DBR cavities exhibiting only bare photonic dispersion (Figures S5 and S6).

The experimental reflectivity spectra of the cavity (Figure 1c) exhibit one feature within the DBR stopband at ca. 2.75 eV. This feature exhibits a flatter dispersion and larger line width than the corresponding cavity photon mode and was thus assigned to the lower polariton. Additionally, the intensity of this feature decreases as a function of the increasing angle. The decreasing intensity profile of the feature as a function of angle is characteristic of the lower polariton and inconsistent with the expected reflectance of a purely photonic cavity mode, which should not exhibit an angle-dependent intensity profile. Spectral cuts of the reflectivity map are included in the Supporting Information for clarity (Figure S3). In order to extract the Rabi splitting, we described the LP dispersion with a coupled-oscillator model of the experimental reflectivity spectra constrained by the empty-cavity dispersion and the PFO exciton energy, as shown in Figure S4 and described in further detail in the surrounding text. The extracted Rabi splitting of 424 meV corresponds in this model to a light-matter coupling strength of 212 meV, comparable to the previously reported value for a similar system of 261 meV.^14^ In highly negatively detuned cavities such as this, the UP can be difficult to detect as it is mostly photonic in character, and the reflectance signal is too weak to observe through the top DBR of high-Q DBR cavities. This effect is common in high-Q organic cavities and poses a challenge for definitive assignment of the strong-coupling regime. However, the UP can be readily detected in similarly structured metallic Fabry–Pérot cavities, giving a Rabi splitting of ca. 1 eV (Figure S5). Moreover, the mode curvature in Figure 1c is distinctly flatter than in equivalent DBR cavities incorporating a nonabsorbing polymer spacer layer (i.e., a purely photonic mode, Figure S6). Combining the optical parameters extracted from Figures S5 and S6, we cleanly reproduce the spectra of our cavity structures using TMM (Figure 1d), both in energetic position and intensity profile, corroborating our assignments of the features.

The microcavity was excited nonresonantly at 3.60 eV (345 nm) at an incident angle of 10° to the cavity normal. In the cavity emission spectrum (Figure 2a), the LP is observed at ca. 2.75 eV, in excellent agreement with the measured cavity reflectivity spectra. Additionally, a feature is observed at ca. 2.39 eV that corresponds to emission from the exciton reservoir through the DBR sideband, termed exciton leakage. Above an absorbed excitation fluence of 30 μJ/cm^2^, the integrated emission exhibits a superlinear increase in intensity. The nonlinear increase in emission intensity is accompanied by a decrease in line width as a function of excitation fluence (Figure 2b,c), as well as a blueshift of the primary LP emission feature (measured from the peak centroid, Figure 2d) and a collapse of the angular emission dispersion around 0 degrees (Figure S7). In inorganic semiconductor microcavities, this blueshift is typically taken as a hallmark of condensation, arising from enhanced polariton–polariton and polariton–exciton interactions as the polariton density is increased.^47–51^ In the case of Frenkel excitons, however, the Bohr radius is considerably smaller, and Frenkel exciton–polariton pair interactions are not expected to contribute significantly to the observed blueshift.^47^ Rather, this blueshift has been attributed to other effects such as Rabi quenching, in which the Rabi splitting is reduced as a result of decreased population of the ground state, and cavity mode energy renormalization resulting from a decrease of the intracavity effective refractive index, for example.^47,52^ Nevertheless, we do observe a blueshift of 2 meV above the onset of nonlinearity that further increases as a function of excitation fluence and, taken together with the nonlinear increase in intensity and decrease in line width, is characteristic of condensation. These spectral signatures agree well with a previous study of similar strong-coupled PFO cavities, where interferometric measurements of long-range spatial coherence were invoked to support the invocation of polariton condensation.^14^ Given the similarity of our cavity system and spectroscopic observables, we find the same process to be active here, which we substantiate further through the appearance of Bose–Einstein population statistics.

**Figure 2:**
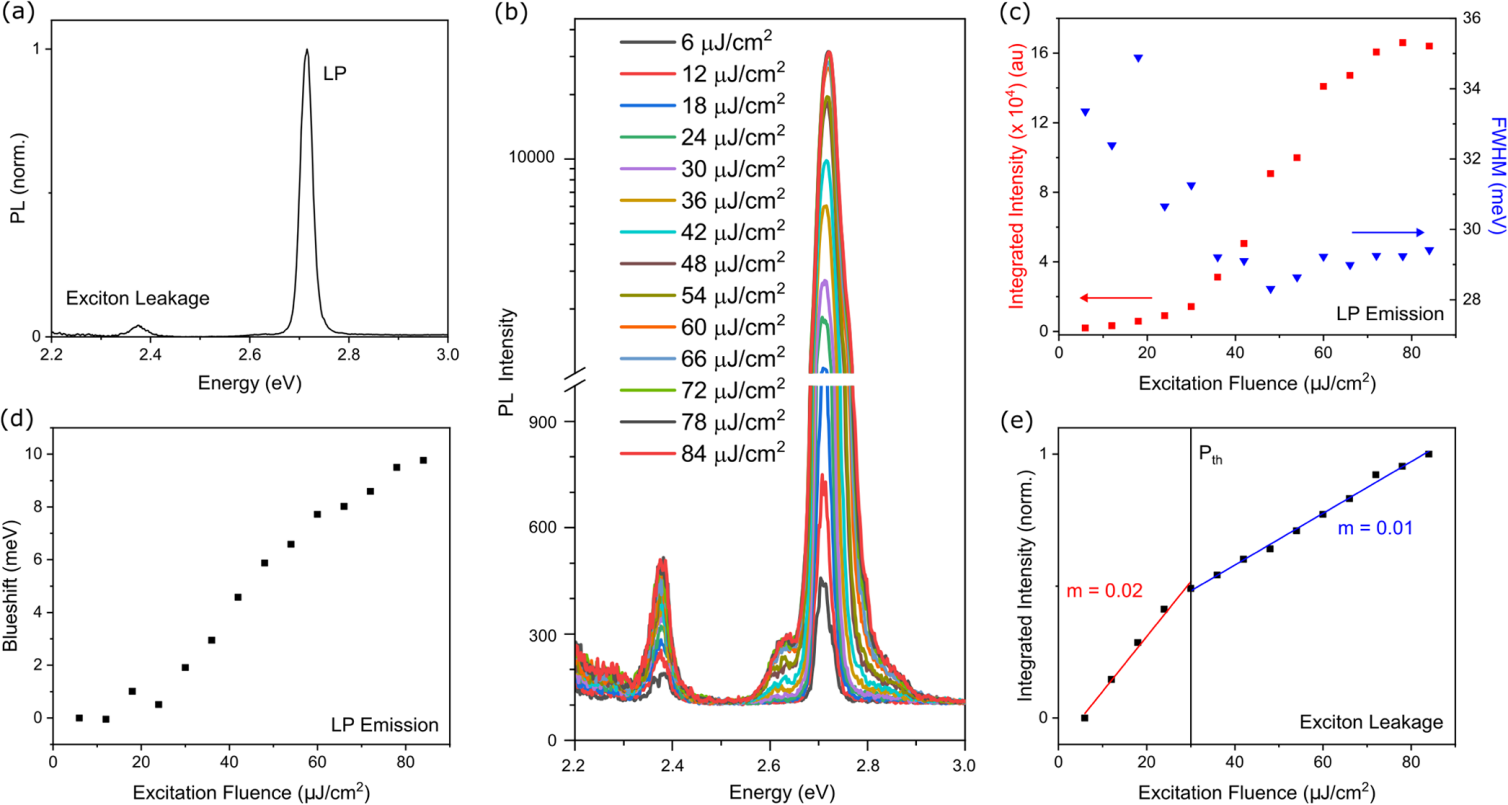
(a) Angularly integrated cavity emission spectrum (0–26°). (b) Cavity emission spectra with increasing excitation fluence. A semilog plot and normalized exciton leakage spectra can be found in Figures S8 and S9. (c) Red: LP emission intensity as a function of excitation fluence; blue: LP emission line width as a function of excitation fluence. (d) LP emission blueshift as a function of excitation fluence. (e) Exciton leakage intensity as a function of excitation fluence. Slope of linear fits given by *m*.

Organic semiconductors exhibit emission with a bandwidth much wider than that of conventional inorganic semiconductors. In this work, the PFO emission extends beyond the DBR stopband (Figure 1a) and allows us to simultaneously probe the behavior of intracavity excitons. Below the stopband, the DBR acts as a filter on the emission from intracavity excitons. Where the DBR sidebands and PFO emission overlap, “exciton leakage” can be observed through regions of high DBR transmission (Figure 2a). This is an advantage unique to the broad emission of organic semiconductors that provides an additional probe into the intracavity photophysics that has not been previously considered, particularly in the context of polariton condensation.

Interestingly, exciton leakage exhibits a different excitation fluence dependence compared to polariton emission. Below the condensation threshold, the exciton leakage increases linearly with the pump fluence. Above the threshold, the slope of this dependence promptly drops by a factor of 2 (Figure 2e). At no point does the spectral shape change (Figure S9), suggesting this emission is always dominated by the same species, as likewise observed in comparable measurements on a bare PFO film (Figure S10). This behavior indicates that an additional loss pathway becomes accessible for the exciton reservoir at the onset of the condensation. The simultaneous increase in the LP population coupled with the increased depopulation of the exciton reservoir is consistent with population transfer from the exciton reservoir to the LP. The exciton leakage is not entirely quenched by the condensate, however, and continues to increase linearly in intensity with a higher excitation power. This suggests that the stimulated scattering from the exciton reservoir to the bottom of the LP dispersion may not be very efficient. Further investigation is needed to understand this effect and the implications it may have on polariton devices involving BEC, such as polariton transistors.^21^

Furthermore, the excitation fluence dependence of the exciton leakage excludes the possibility that the feature assigned to the LP is simply the PFO emission filtered through the cavity photon mode. This is a particularly important point in the case of PFO, as PFO exhibits amplified spontaneous emission (ASE) of the 0–1 vibronic replica at approximately 2.75 eV,^45^ resonant with the LP of the cavity in Figure 1 at 0°. We observe the same nonlinear behavior in a slightly thinner cavity, fabricated such that the LP emission sits approximately 50 meV above the ASE energy (Figure S11). Still, unambiguously distinguishing polariton condensation from photon lasing in strongly coupled microcavities remains a challenge in the literature, as they exhibit many of the same observables.^35^ In rare cases, an additional threshold can be observed when the system is driven out of the strong coupling regime and photon lasing occurs, clearly differentiating the two effects.^16,39,53^ This behavior is typically inaccessible with organic materials, as the material often degrades before the onset of photon lasing. We can exclude photon lasing on the basis of: (1) additional depletion of the exciton reservoir coupled with increased LP population above the condensation threshold (Figure 2c,e), and (2) the observed crossover from Maxwell–Boltzmann statistics to Bose–Einstein statistics (see above).

In order to probe the thermalization behavior of the polariton condensate, relative polariton populations were derived from the measured emission spectra by normalization of the spectra by the photon fraction |*C*(*k*_||_)|^2^ obtained from a coupled oscillator model of the cavity reflectivity (Figure S4) in a similar method to that employed by Sun et al.,^54^ Satapathy et al.,^17^ and Hakala et al.^43^ Below the condensation threshold, we can describe the polariton population distribution well with a simple exponential decay as a function of the energetic separation from the LP minimum (Figure 3a, black symbols). This behavior reflects the Maxwell–Boltzmann statistics of the exciton reservoir, which populates the LP. Above the condensation threshold, the polariton population distributions were fitted using the equilibrium Bose–Einstein distribution of eq 1, as shown for select excitation fluences in Figure 3a (fit residual and additional fluences presented in Figures S12 and S13). This behavior is in contrast to the polariton populations calculated from a cavity that did not exhibit BEC (Figure S14). This cavity was prepared with a similar structure to the one shown in Figures 1 and 2, with DBRs prepared in the same fabrication cycle and an ∼100 nm active layer of PFO spin-coated under identical conditions, leading to a similar Rabi splitting of ca. 400 meV. The principal difference is that the LiF spacer layer was omitted, resulting in a less negative detuning with the LP minimum blue-shifted to 2.90 eV. Over our accessible range, the LP emission intensity in this cavity simply scales linearly with excitation fluence. Treating the resulting spectra similarly to Figure 3a, we find that the polariton population distributions are well-described as monoexponential at all measured excitation fluences (Figure S14). They exhibit no signatures of the Bose–Einstein statistics.

**Figure 3:**
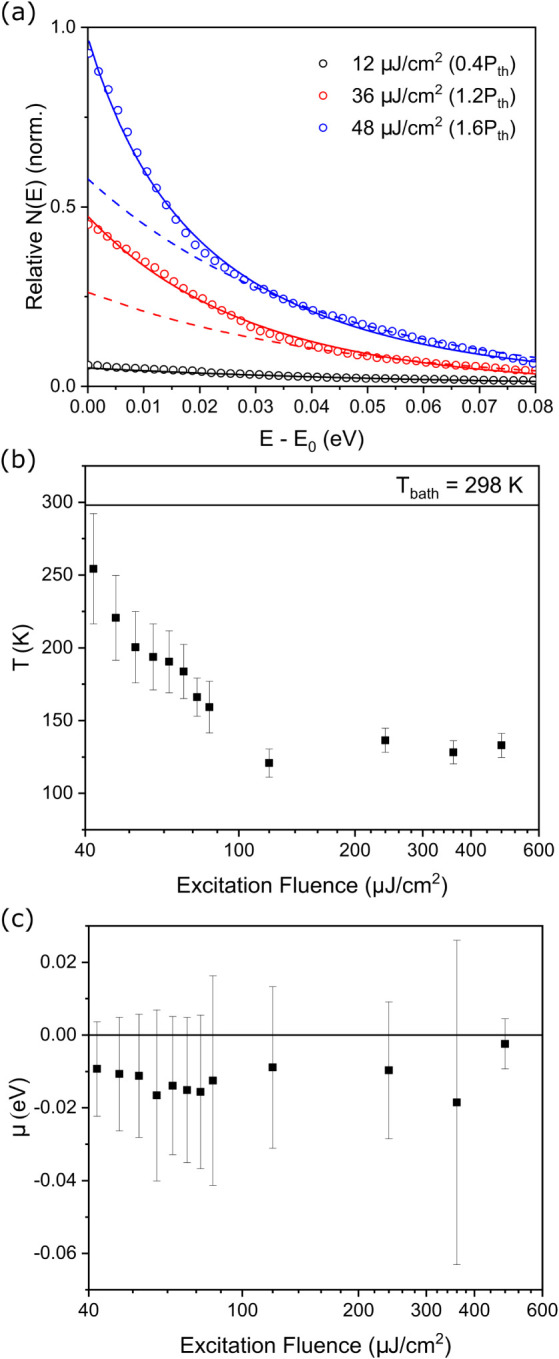
(a) Deviation from Maxwell–Boltzmann statistics of the measured polariton distributions above and below the condensation threshold. Solid lines are Bose–Einstein fits; dashed lines are Maxwell–Boltzmann (monoexponential) fits to the Boltzmann tail extrapolated to zero. The *x*-axis is scaled linearly with respect to the LP emission maximum E_0_ for clarity, as the extracted temperature and chemical potential depend only on the curvature of the distribution, not the energy. (b) Average temperature of the polariton gas as a function of excitation fluence. (c) Average chemical potential of the polariton gas as a function of excitation fluence.

From fitting the population distributions, we extract the effective temperature and chemical potential of the polariton gas (Table S1). After the onset of condensation, the average temperature of the polariton gas decreases below the bath temperature and continues to decrease until about 130 K, after which it remains effectively constant within error (Figure 3b). This stimulated cooling of the polariton gas below the bath temperature itself is a distinct feature of this class of nonequilibrium BEC, as predicted by Shishkov et al. from their analytical expression for the density matrix.^37^ While the microscopic mechanism of this sublattice thermalization is unexplained, it serves as a powerful hallmark of nonequilibrium BEC physics. The relative error in the chemical potential is higher than that in the effective temperature, precluding us from drawing any significant conclusion from the behavior of the chemical potential alone. However, the chemical potential is negative, small in magnitude, and generally increases toward zero as the polariton density is increased (Figure 3c) and thus does not contradict the expected behavior of the chemical potential of a nonequilibrium exciton-polariton BEC, noisy though it is.

## Conclusions

In summary, we conclude that the experimental observations above are best described by a nonequilibrium BEC, and that the phenomenon of Bose–Einstein condensation is thus a more general phenomenon, of which condensation at thermodynamic equilibrium is a specific case. The temperature dependence of the polariton gas is in excellent qualitative agreement with that predicted by Shishkov et al.,^37^ and observation of such behavior supports the description of such systems in terms of Bose–Einstein condensation. We expect the thermalization behavior of such systems to hold implications for phenomena such as room-temperature polariton superfluids, for example. Interestingly, the observation of the effect of condensation on the exciton reservoir population—as observed through the exciton leakage—demonstrates a unique advantage of organic materials in understanding the fundamental photophysics of polariton BECs, providing an additional probe into the relevant population dynamics inaccessible to more traditional semiconductors. This effect is of particular importance when trying to differentiate polariton BEC from photon lasing, as they exhibit many of the same observables. The change in slope of the excitation fluence dependence of the exciton leakage above the condensation threshold coupled with the crossover from Maxwell–Boltzmann to Bose–Einstein statistics should serve as a robust hallmark of condensation in organic materials. We expect that the change in slope of the exciton leakage fluence dependence below and above threshold will serve as a metric to evaluate the efficiency of bosonic stimulation/BEC formation, since it reports on excitons that remain in the reservoir rather than scattering into the LP/BEC. Collected across a library of systems, this behavior may provide guidance to enhance the efficiency of condensation to enable new low-power applications of room-temperature organic polariton devices. Similarly, the dependence of the observed thermalization behavior on material properties—beyond the fast thermalization of Frenkel excitons intrinsic to organic semiconductors—and cavity structure remains an open question. It will be instructive to apply our approach to a wider family of organic BECs in order to clarify which factors are relevant to the microscopic mechanism of polariton thermalization in organic semiconductors.

## Supplementary Material



## Notes

Additional spectroscopic characterization, fitting parameters, and reference cavities (PDF)
