# Supplementary material for: Stimulated Cooling of a Frenkel Exciton-Polariton Bose–Einstein Condensate at Room Temperature
Source: ACS Photonics. 2026 Apr 22;13(9):2483–9. doi: 10.1021/acsphotonics.5c03069 (PMC13154354; doi:10.1021/acsphotonics.5c03069)
Supplement: Supplementary file 1 [file ph5c03069_si_001.pdf]

Supplemental Material for "Stimulated Cooling of a Frenkel Exciton-Polariton Bose-Einstein Condensate at Room Temperature"

Thomas M. Khazanov, Cora A. Noble and Andrew J. Musser*

Department of Chemistry and Chemical Biology, Cornell University, Ithaca, New York 14853, USA

*Correspondence should be addressed to ajm557@cornell.edu

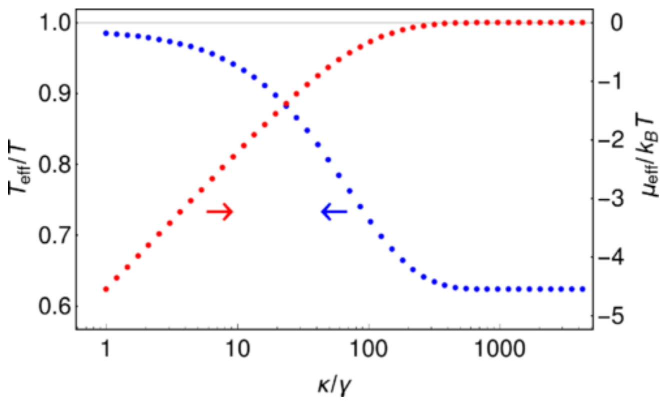

Figure S1. Approximation of the average polariton population in a nonequilibrium BEC by the equilibrium Bose-Einstein distribution as described in ref. 36. (Blue) The ratio of the effective temperature of polaritons to the reservoir temperature as a function of the rate of incoherent pumping. (Red) Effective chemical potential of polaritons as a function of the rate of incoherent pumping. Reprinted with permission from Shishkov et al., Phys. Rev. Lett. 128, 065301-5 (2022). Copyright 2022 by the American Physical Society. http://dx.doi.org/10.1103/PhysRevLett.128.065301.

Peak Analysis

Data Set:[Book1]Sheet1!B Date:01-Apr-26

BaseLine:Constant

Chi^2=5.76020E-01 Adj. R-Square=9.72442E-01 # of Data Points=25001

SS=1.43988E+04 Degrees of Freedom=24997

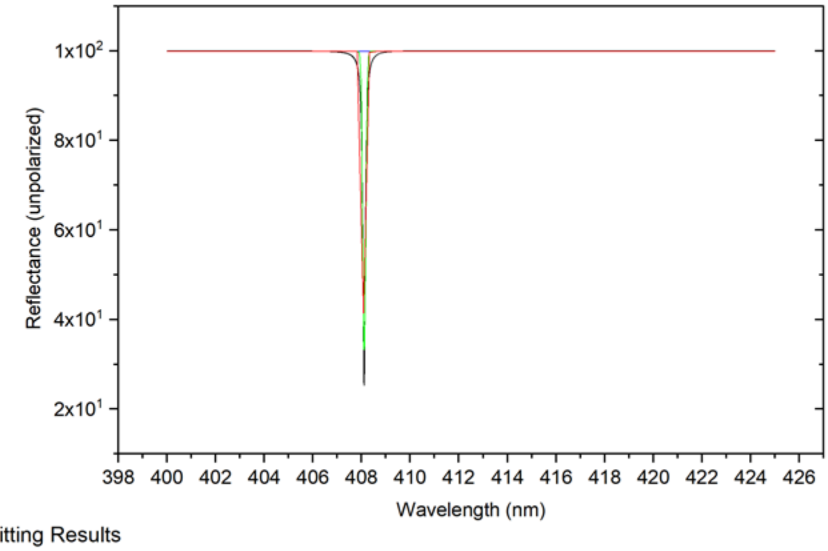

Fitting Results

Peak Index	Peak Type	Area Intg	FWHM	Max Height	Center Grvty	Area IntgP
1	Gaussian	-14.81514	0.15316	-66.70142	408.11427	-100

Figure S2. Gaussian fit of the computed cavity mode at normal incidence via transfer matrix modeling of the cavity corresponding to Fig. 3 in the main text. From this we obtain a Q-factor of 2664.

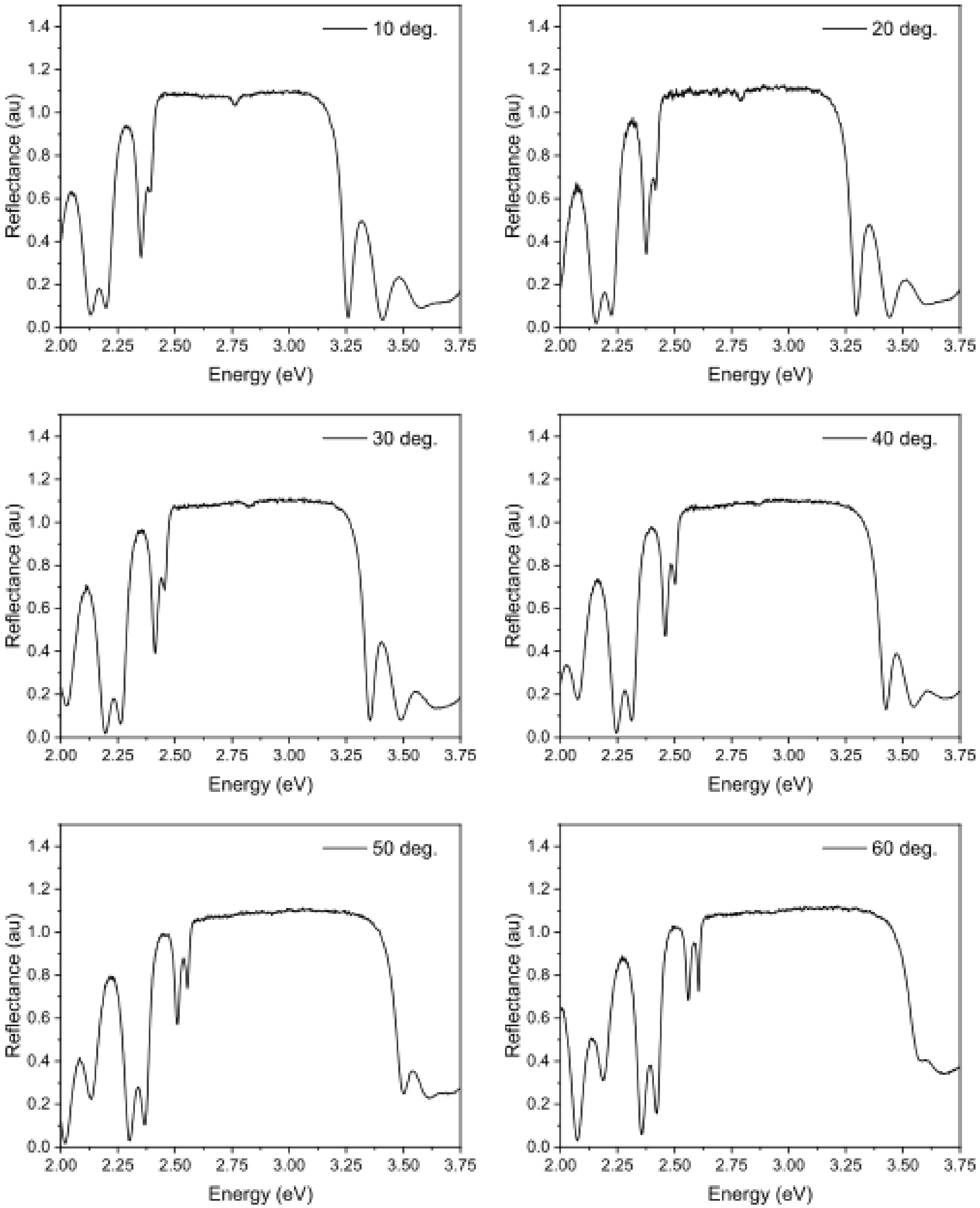

Figure S3. Spectral cuts of the experimental reflectivity map in Fig. 2 of the main text.

The polariton coupled oscillator model is given by

$$\begin{pmatrix} E_\gamma(\theta) & \hbar\Omega/2 \\ \hbar\Omega/2 & E_x \end{pmatrix} \begin{pmatrix} \alpha_\gamma(\theta) \\ \alpha_x(\theta) \end{pmatrix} = E_P \begin{pmatrix} \alpha_\gamma(\theta) \\ \alpha_x(\theta) \end{pmatrix},$$

in which the energy of the cavity photon mode as a function of angle is given by $E_\gamma(\theta)$, the exciton energy by E_x, polariton mixing coefficients (*vide infra*) describing the photon and exciton fraction of the polariton as $\alpha_\gamma(\theta)$and $\alpha_x(\theta)$, and the polariton energy by E_P. Diagonalization yields

$$E_P = \frac{{E_\gamma}^2 + {E_x}^2}{2} \pm \frac{1}{2}\sqrt{\left(E_\gamma + E_x\right)^2 + (\hbar\Omega)^2},$$

where the negative solution corresponds to the lower polariton and the positive solution to the upper polariton with mixing coefficients

$${\alpha_\gamma}^2 = \frac{E_\gamma - E_P}{E_\gamma + E_x - 2E_P},$$

$${\alpha_x}^2 = 1 - {\alpha_\gamma}^2.$$

The eigenstate equation was solved numerically in MATLAB for Ω as follows. First, transfer matrix modeling of the experimental angle-dependent cavity reflectance spectra was performed. The reflectance spectra were fit to a Gaussian function at each angle to yield a vector containing the angle and centroid of the Gaussian. This was fit to a polynomial to yield a closed-form expression describing the measurable LP dispersion. The LP reflectance from transfer matrix modeling was used instead of the experimentally measured LP reflectance as our goniometer can only access angles as low as 9 degrees. While closed-form solutions for the cavity photon mode dispersion exist for simple cavities, for complex structures such as those in this work, it is preferable to directly compute the cavity mode numerically through transfer matrix modelling. The above process was repeated to generate an expression for the cavity mode energy as a function of angle. From here, the value of Ω can be optimized to minimize the difference between the calculated E_P for that value of Ω and the value obtained from transfer matrix modeling at each angle. Final adjustment of the model was performed manually. The resulting fit of the LP dispersion is presented in Figure S4.

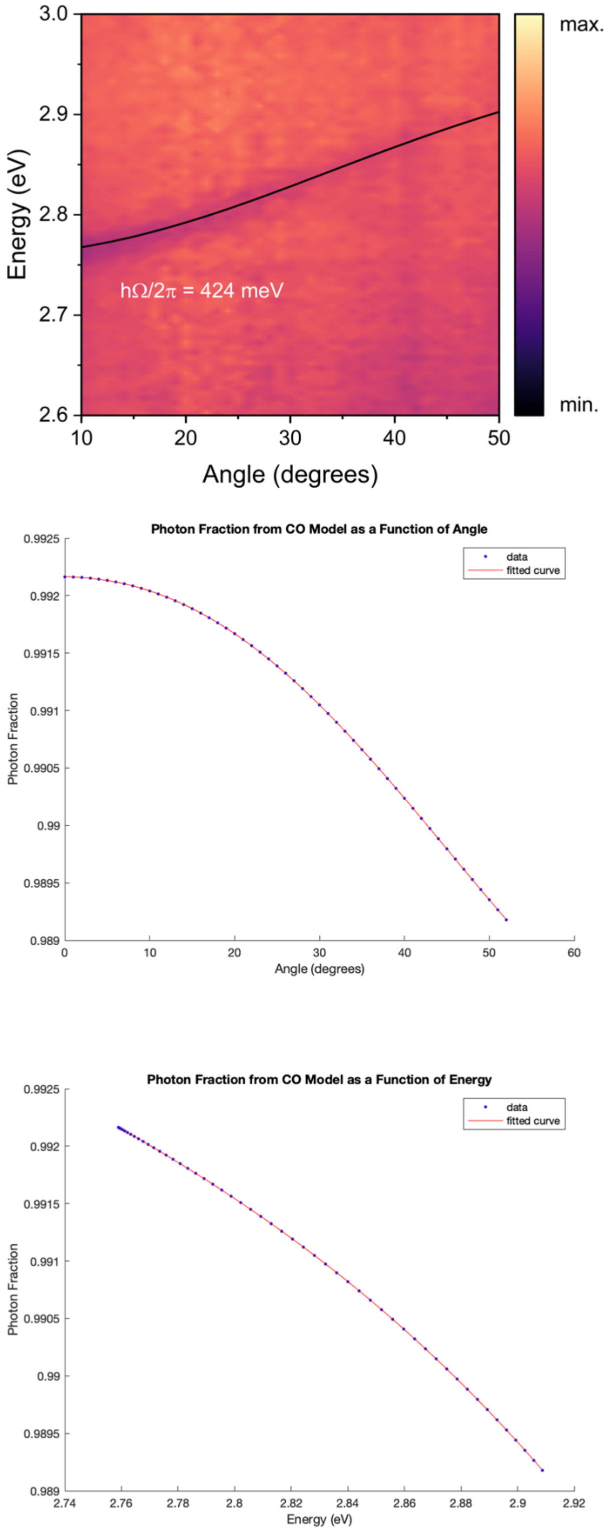

Figure S4. Coupled oscillator model of the experimental reflectivity and extracted photon Hopfield coefficient as a function of angle and energy.

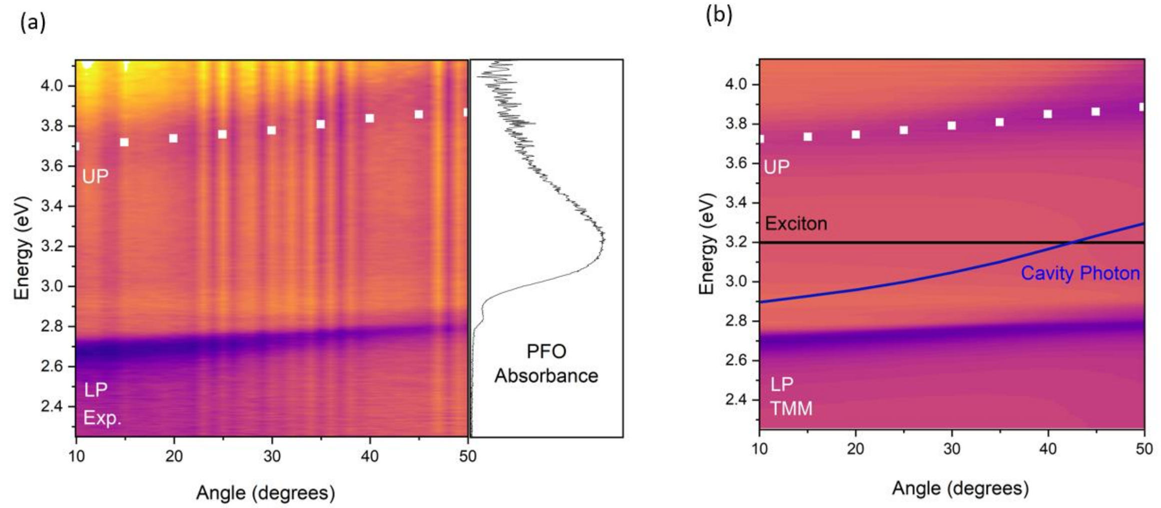

Figure S5. Angle-resolved reflectance for strongly coupled Al-PFO-Al cavities. (a) Contour map of the angle-resolved reflectance spectra that exhibits both LP and UP features (left), with evident anti-crossing around the PFO film absorption band (right). (b) Transfer matrix modeling of the metallic cavity reflectance in (a) with the PFO exciton peak (black) and the cavity mode (blue) indicated. UP dispersion indicated by white squares for clarity. These measurements are used to benchmark the optical constants for the PFO layer used in the condensation structures reported in the main text.

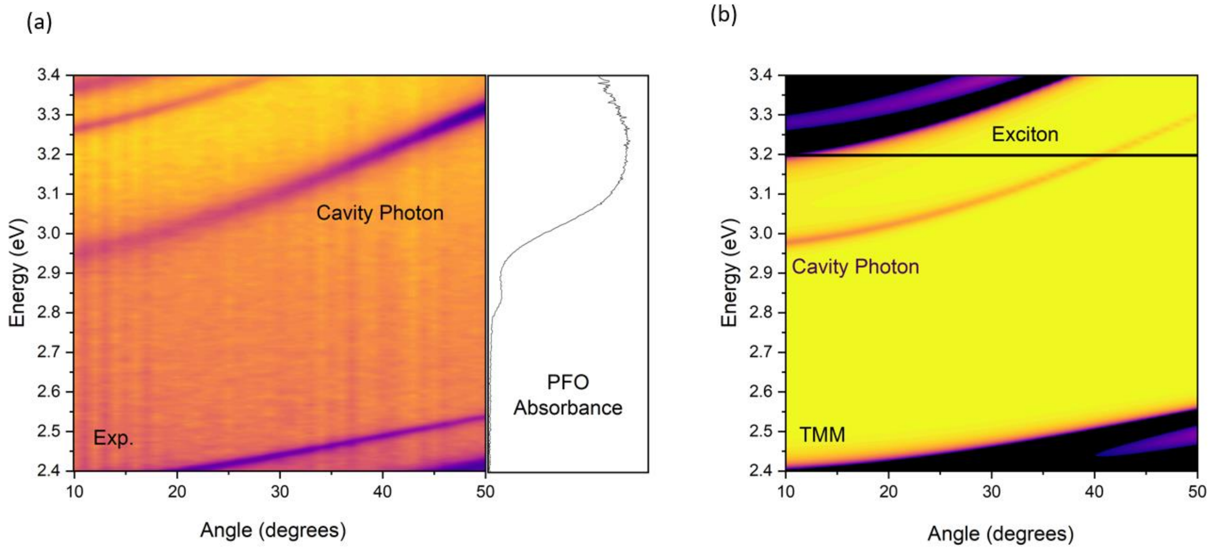

Figure S6. Lower-Q DBR cavities with no PFO absorbance. (a) Angle-resolved reflectance of a low-Q DBR cavity (2.5-pair top, 9.5-pair bottom mirrors, same composition as in the main text) with similar active layer thickness to the structure reported in the main text, but no absorbing PFO layer. The cavity photon mode exhibits typical dispersion, with no evidence of anti-crossing. The PFO absorbance spectrum is plotted alongside as a reference for where polaritonic effects would be expected to occur if there were an absorbing layer. (b) Transfer matrix modeling of the structure in (a), with the PFO exciton (black) indicated for reference. These measurements are used to benchmark the optical constants used to describe the condensation structures reported in the main text, and to determine where the photonic mode lies in main-text Fig. 1d.

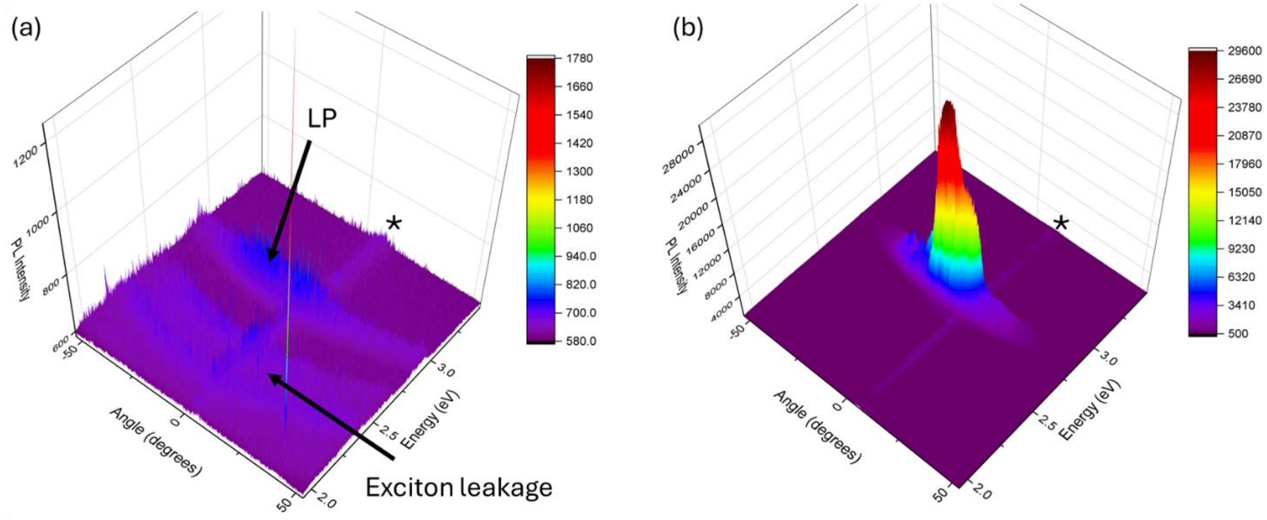

Figure S7. Fourier-plane imaging of the cavity emission in the cavity that exhibits BEC (a) below and (b) above the condensation threshold. Both LP emission and exciton leakage signals through the angle-dependent DBR sidebands can be observed at lower excitation densities. The angle-independent signal indicated by * is due to a damaged pixel.

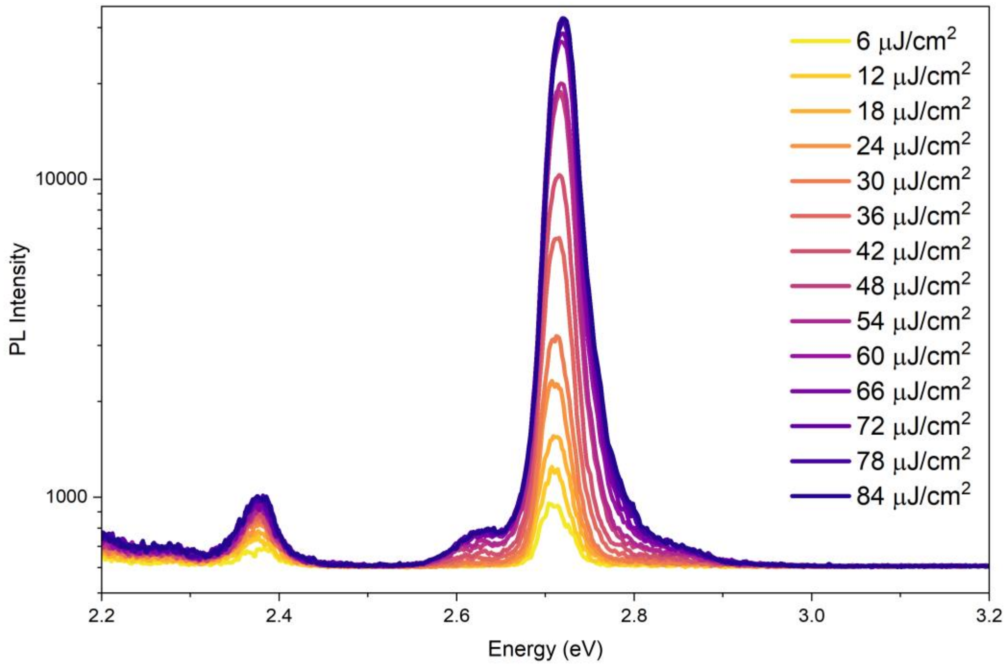

Figure S8. Fluence-dependent emission spectra of the cavity corresponding to Fig. 3 in the main text.

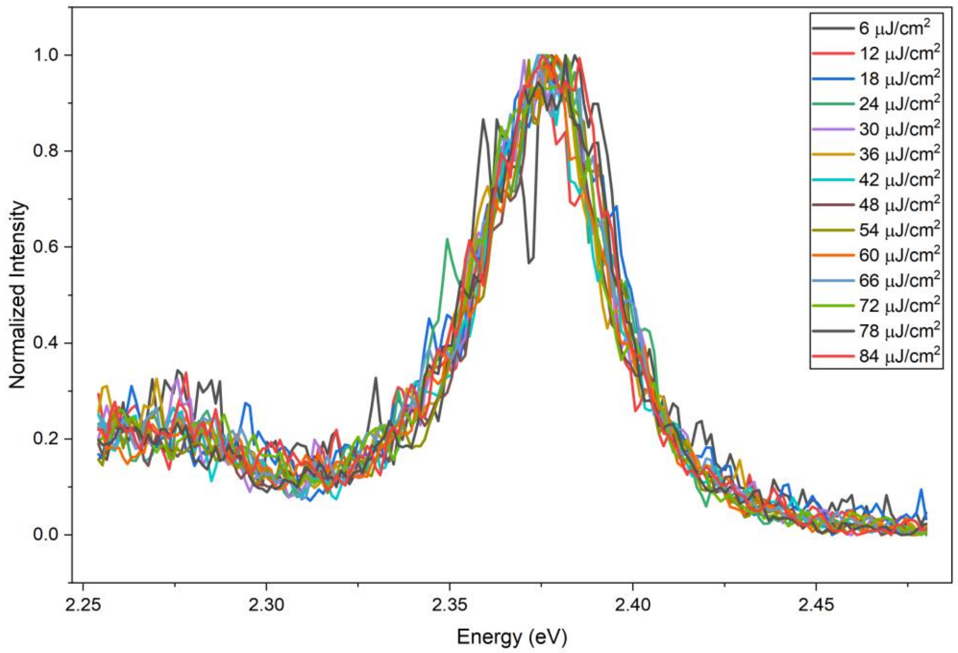

Figure S9. Expansion of the exciton leakage in Figure S8. The normalized spectra exhibit no observable change in lineshape as a function of excitation fluence under the recorded excitation fluences.

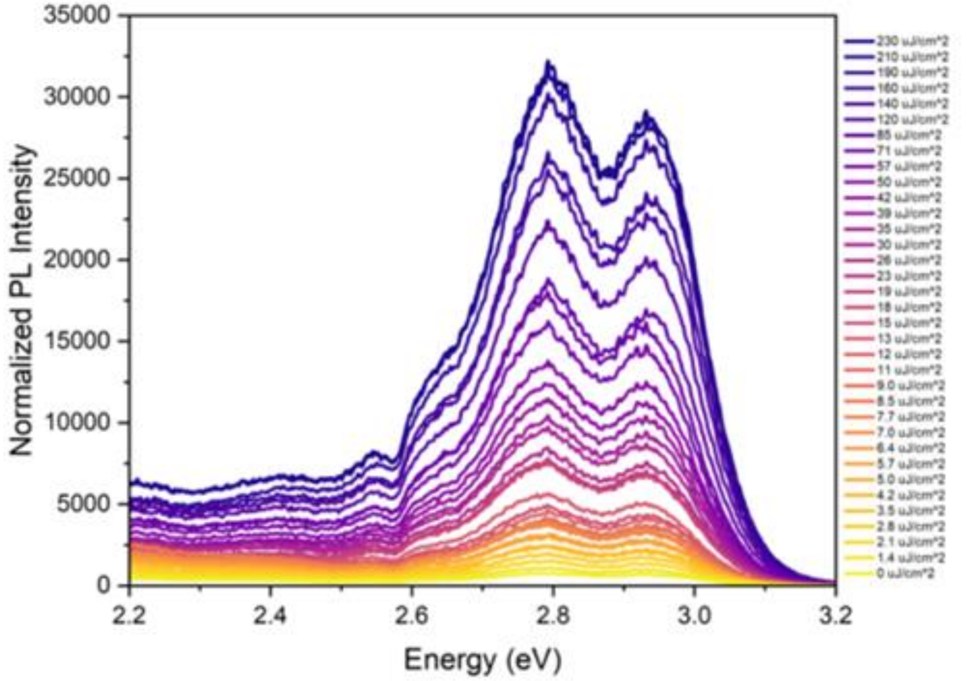

Figure S10. Normalized emission spectra of a bare PFO film over a wider range of excitation densities than applied in cavity measurements. The exciton emission exhibits no change in lineshape or energy as a function of excitation fluence.

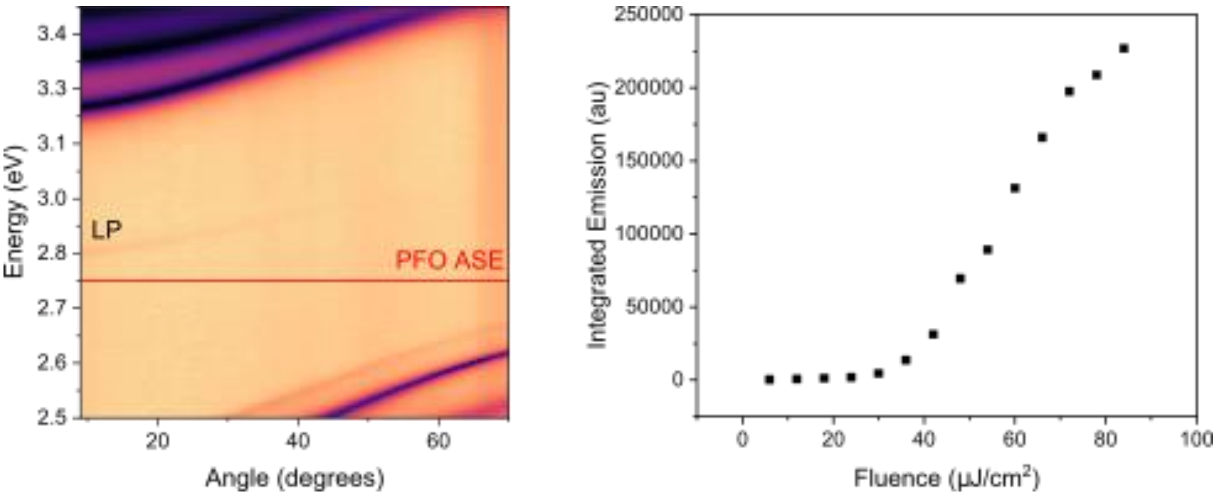

Figure S11. Experimental reflectivity of a cavity that exhibits the LP above PFO ASE energetically and a nonlinear increase in emission intensity as a function of excitation fluence consistent with condensation.

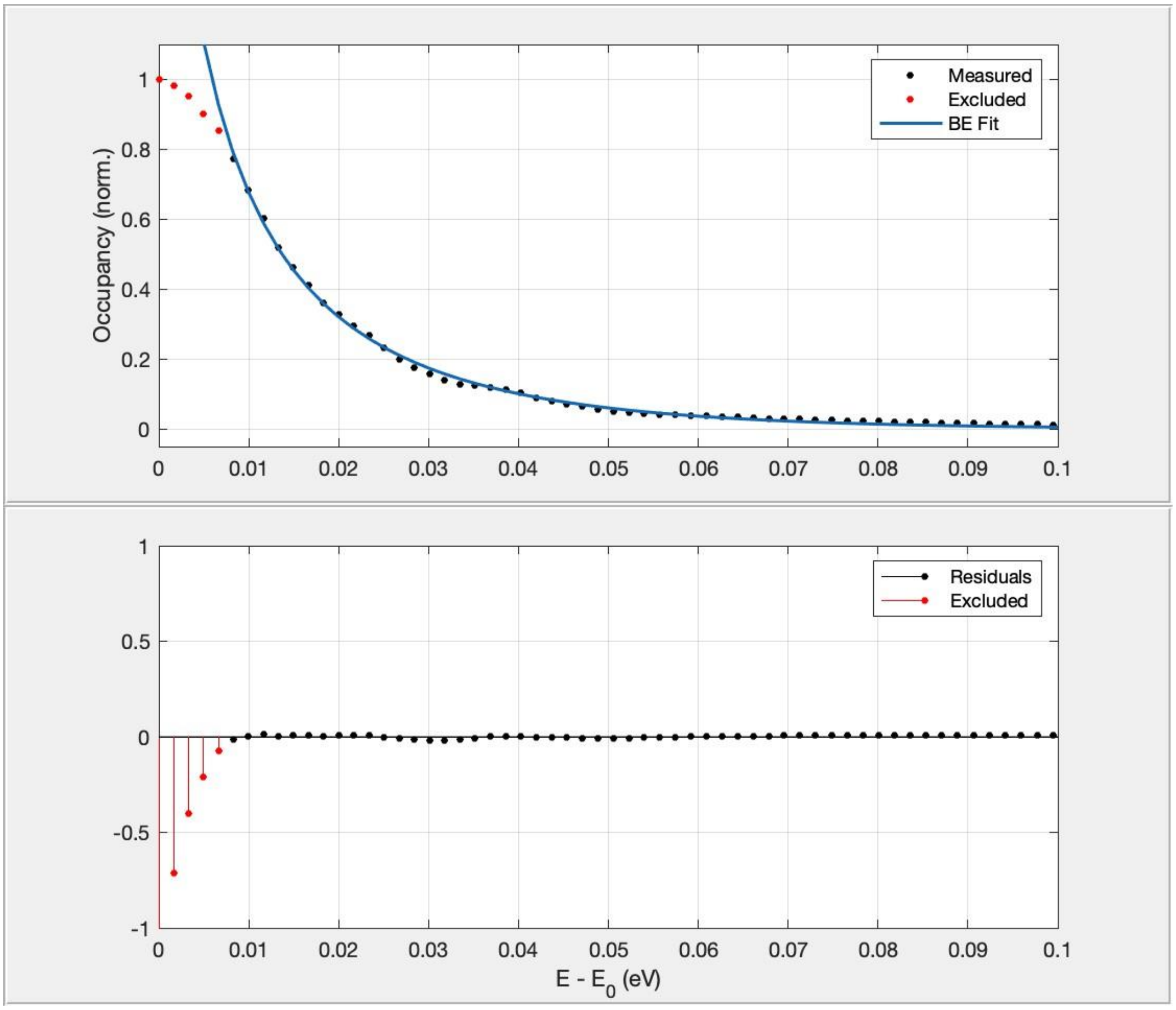

Figure S12. Bose-Einstein fit to calculated population distribution at excitation fluence 240 μJ/cm^2.

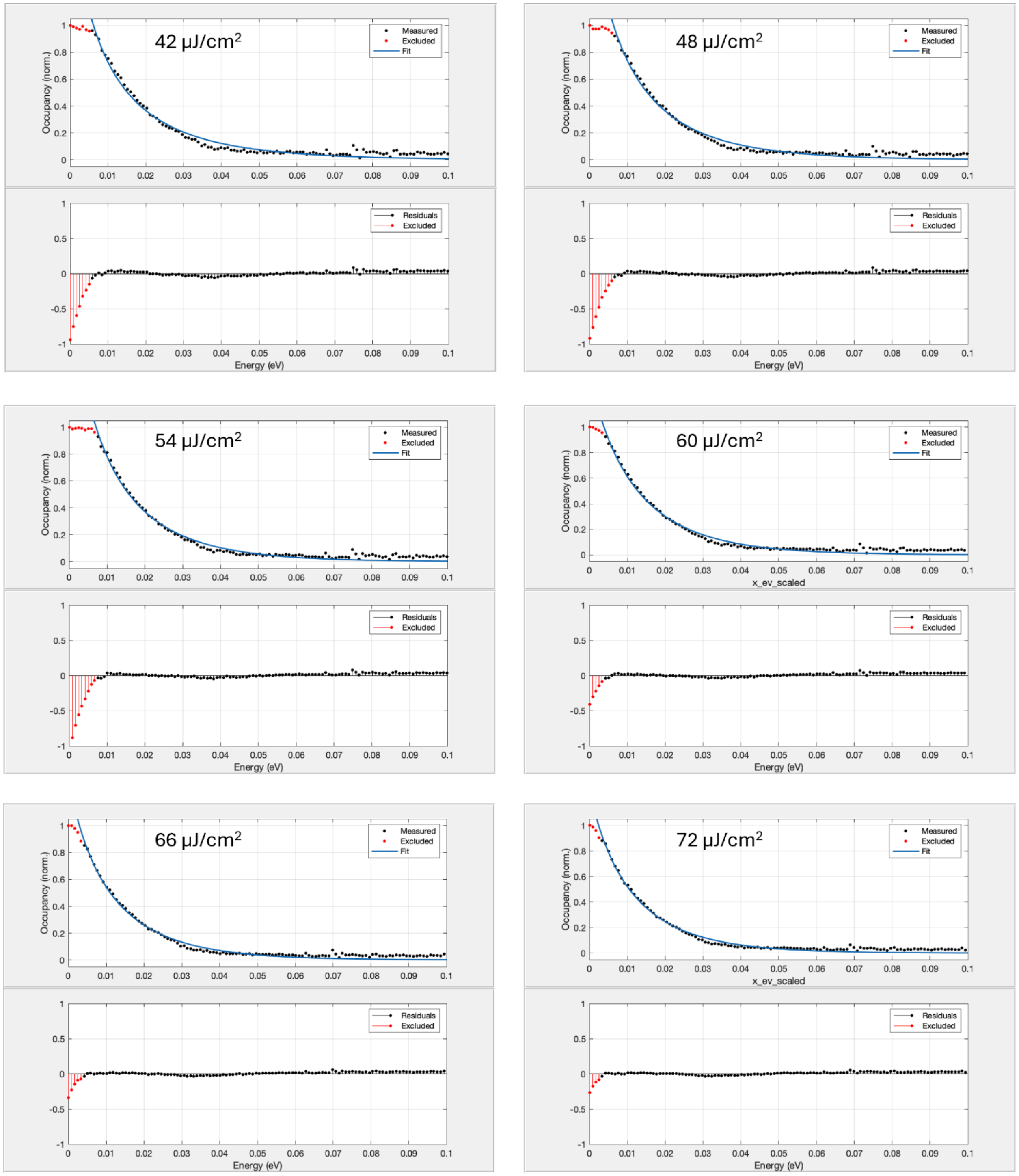

Figure S13. Representative Bose-Einstein fits to calculated population distribution as in Fig. S12 across a range of excitation fluences.

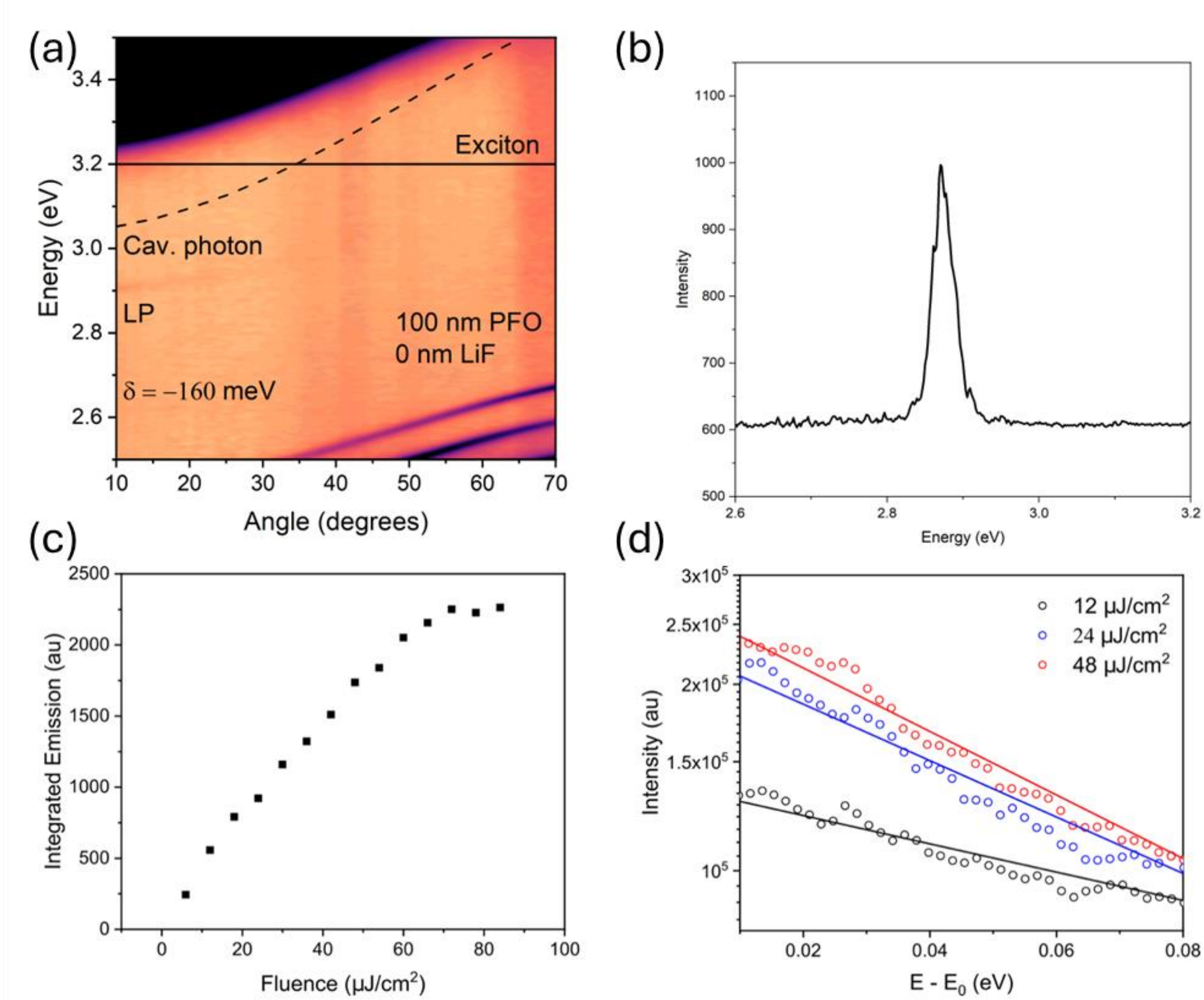

Figure S14. (a) Angle-resolved reflectance of a cavity that did not exhibit condensation. The corresponding bare-cavity photon dispersion and bare-film, exciton peak energy are superimposed as dashed and solid lines. The cavity had a ca. 100 nm spin-coated PFO active layer and no LiF spacer, yielding an exciton-photon detuning of -160 meV. (b) Corresponding PL spectrum of the cavity. (c) Fluence-dependent PL increases linearly with excitation density until saturation at high laser powers. (d) The calculated population distributions of the emission tail, extracted similarly as for BEC-forming cavity, exhibit monoexponential behavior consistent with Maxwell-Boltzmann statistics.

Table S1. Extracted parameters from Bose-Einstein fit of the calculated population distributions and goodness of fit.

Fluence ($\mu J/cm^2$)	T (K)	T Lower Bound	T Upper Bound	μ (eV)	μ Lower Bound	μ Upper Bound	R-squared
42	254.2638	254.2638	292.1127	-0.0093	-0.0130	-0.0056	.9244
48	220.5968	220.5968	249.7636	-0.0107	-0.0156	-0.0059	.9371
54	200.4222	200.4222	225.0290	-0.0112	-0.0169	-0.0055	.9445
60	193.7291	193.7291	216.443	-0.0166	-0.0235	-0.0096	.9482
66	190.4102	190.4102	211.6657	-0.0139	-0.0190	-0.0088	.9546
72	183.7115	183.7115	202.3443	-0.0151	-0.0200	-0.0101	.9625
78	166.0867	166.0867	179.2762	-0.0156	-0.0211	-0.0100	.9769
84	159.2113	159.2113	176.9450	-0.0125	-0.0288	-0.0023	.9537
120	120.8202	120.8202	130.4927	-0.0089	-0.0222	-0.0045	.9768
240	136.4651	136.4651	144.7906	-0.0097	-0.0188	-6E-4	.9870
360	128.2329	128.2329	136.1611	-0.0185	-0.0446	0.0075	.9864
480	132.9397	132.9397	141.2322	-0.0024	-0.0069	0.0021	.9870
